# Impact of Different Oseltamivir Regimens on Treating Influenza A Virus Infection and Resistance Emergence: Insights from a Modelling Study

**DOI:** 10.1371/journal.pcbi.1003568

**Published:** 2014-04-17

**Authors:** Laetitia Canini, Jessica M. Conway, Alan S. Perelson, Fabrice Carrat

**Affiliations:** 1 INSERM, UMR_S 1136, Institut Pierre Louis d'Epidémiologie et de Santé Publique, Paris, France; 2 Sorbonne Universités, UPMC Univ Paris 06, UMR_S 1136, Institut Pierre Louis d'Epidémiologie et de Santé Publique, Paris, France; 3 Theoretical Biology and Biophysics, Los Alamos National Laboratory, Los Alamos, New Mexico, United States of America; 4 Assistance Publique Hôpitaux de Paris, Hôpital Saint Antoine, Paris, France; University of California San Diego, United States of America

## Abstract

Several studies have proven oseltamivir to be efficient in reducing influenza viral titer and symptom intensity. However, the usefulness of oseltamivir can be compromised by the emergence and spread of drug-resistant virus. The selective pressure exerted by different oseltamivir therapy regimens have received little attention. Combining models of drug pharmacokinetics, pharmacodynamics, viral kinetics and symptom dynamics, we explored the efficacy of oseltamivir in reducing both symptoms (symptom efficacy) and viral load (virological efficacy). We simulated samples of 1000 subjects using previously estimated between-subject variability in viral and symptom dynamic parameters to describe the observed heterogeneity in a patient population. We simulated random mutations conferring resistance to oseltamivir. We explored the effect of therapy initiation time, dose, intake frequency and therapy duration on influenza infection, illness dynamics, and emergence of viral resistance. Symptom and virological efficacies were strongly associated with therapy initiation time. The proportion of subjects shedding resistant virus was 27-fold higher when prophylaxis was initiated during the incubation period compared with no treatment. It fell to below 1% when treatment was initiated after symptom onset for twice-a-day intakes. Lower doses and prophylaxis regimens led to lower efficacies and increased risk of resistance emergence. We conclude that prophylaxis initiated during the incubation period is the main factor leading to resistance emergence.

## Author Summary

Oseltamivir is currently the most commonly used drug against influenza but the emergence and spread of oseltamivir-resistant virus is threatening its usefulness. A previously published study quantified the risk of drug-resistance emergence and spread. In this work we investigate under what conditions drug-resistance is likely to occur and how we can mitigate it. For this purpose, we simulated populations of influenza-infected subjects under different treatment conditions varying drug dose, intake frequency and duration of therapy. We used an approach that mimics the randomness of drug-resistance emergence and allowed for between-subject variability. We measured the effect of treatment on reducing infection and symptoms and on drug-resistance emergence. We found that for subjects starting oseltamivir during the influenza incubation period, the risk of resistance emergence is dramatically increased. Thus, our findings suggest that standard prophylaxis should only be used after exclusion of an influenza infection in the incubation period by use of a rapid test. If existing infection cannot be excluded, then prophylaxis should be done with increased dose, intake frequency and duration in order to avoid emergence of drug-resistant strains and to preserve oseltamivir efficacy.

## Introduction

Besides influenza vaccination, neuraminidase inhibitors are currently the most effective pharmaceutical intervention recommended to reduce the burden of seasonal or pandemic influenza [1], [2], [3], [4], [5], [6]. Two neuraminidase inhibitors are widely marketed: nebulised zanamivir (Glaxo Wellcome) and oral oseltamivir (Hoffmann-La Roche). These drugs block the release of influenza virus from infected host cells and hence reduce the spread of infection in the respiratory tract [7]. Oseltamivir has been stockpiled in many countries for pandemic preparedness and is the most frequently used neuraminidase inhibitor worldwide [8]. Oseltamivir therapy accelerates the time to alleviation of influenza-like illness and post-exposure prophylaxis with oseltamivir reduces secondary transmission of influenza [9]. However, oseltamivir effectiveness strongly depends on the delay between infection (or onset of symptoms) and the first antiviral intake [10]. Oseltamivir effectiveness can also be compromised by the emergence and further spread of drug-resistant viruses such as the H275Y mutant strain [11]. Most resistant isolates emerge during post-exposure prophylaxis [12] or under a curative regimen in subjects with intense or prolonged viral shedding, such as children [13], [14] or immunocompromised persons [15], [16], [17].

The interactions between time of infection, first oseltamivir intake, dose regimen, and host response to infection are complex with respect to symptoms, virological efficacy and emerging resistance. Moreover, the dynamics of influenza infection is highly variable between subjects [18] and the pharmacokinetics of oseltamivir leads to large concentration variations over time [19], which can lead to variable efficacy at the individual level. To the best of our knowledge, the between-subject variability and the effect of oseltamivir pharmacokinetics have never been studied in detail. Here we explore these interactions *in silico*, using a hybrid deterministic/stochastic adaptation of the combination of a pharmacokinetic (PK) model of oseltamivir [19] and a virus kinetic/symptom dynamic (VKSD) model previously fitted to data on experimental human infection [18]. We provide an integrative framework to assess antiviral therapy by simultaneously taking into account random mutations, oseltamivir efficacy, and selective pressure.

## Materials and Methods

### Structural equation modelling

#### Oseltamivir pharmacokinetics

**Figure 1:**
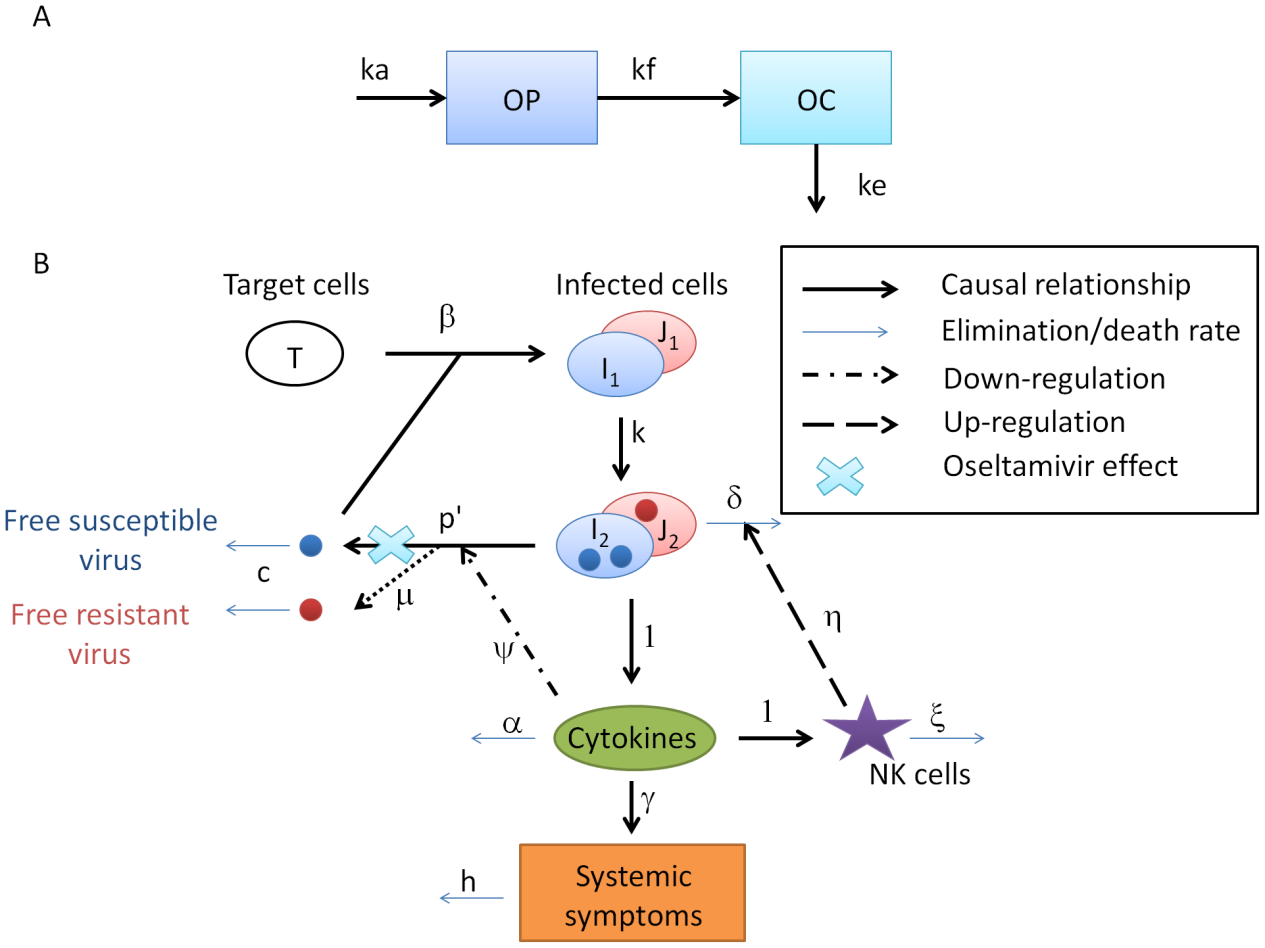
A. Graphical presentation of the PK model of oseltamivir. Oseltamivir phosphate (OP) is absorbed at rate 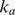
and converted into oseltamivir carboxylate (OC) at rate 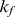. OC is eliminated at rate 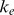. B. Graphical presentation of the viral kinetic symptom dynamic (VKSD) model with the effect of emerging resistance and oseltamivir therapy. Free virus infects target epithelial cells, which become infected cells not yet producing virus, 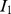 and 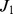, which transition at rate 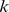 into productively infected cells, 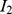 and 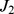. These latter cells produce free virus and lead to the production of pro-inflammatory cytokines, either directly or via activation of macrophages or dendritic cells. Pro-inflammatory cytokines reduce the virus production rate, activate natural killer (NK) cells, and induce systemic symptoms. NK cells kill infected cells. Resistant virus can emerge during replication, infect target cells and induce the VKSD cycle. Oseltamivir acts by blocking the release of free viruses. 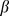 is the target cell infection rate, 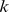 is the transition rate from 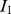 to 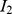, 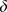 is the mortality rate of infected cells, 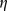 is the effect of NK cells on infected cells, 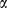 is the cytokine clearance, 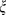 is the mortality rate of NK cells, 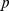 is the rate of virus production by 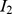, 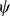 is the effect of cytokines on 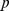, and 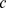 is the virus clearance. 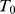, the initial number of target cells in the upper respiratory tract, was set to 4×10^8^. We set the cytokine and NK cell production rates to 1, as it changes only the units in which the early immune response is measured and does not lead to a loss of generality.

The pharmacokinetics of oseltamivir were modeled as functions of the prodrug, oseltamivir phosphate (OP), and its active-drug metabolite, oseltamivir carboxylate (OC). We used a 2-compartment model with one compartment for OP distribution and one for OC distribution (Fig. 1A) [20].

The system of ordinary differential equations (ODEs) used is:
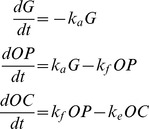
(1)where 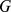 is the depot compartment out of which the drug is absorbed, 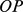 is the prodrug concentration and 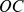 is the active-drug metabolite concentration. 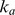 is the absorption rate, 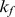 is the conversion rate from OP to OC and 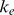 is the elimination rate. The initial conditions are 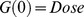, 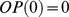, 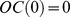. Dose was added in compartment 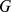 at interval of 8, 12 or 24 hr depending on the regimen. This system was solved analytically (Text S1). Since oseltamivir targets virus release from infected cells, we modelled the drug action by modifying the viral production rate with a time-dependent drug efficacy parameter 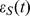. The *OC* concentration is used to define the efficacy of the treatment, 
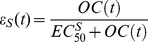
; 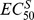 is the OC concentration providing 50% inhibition of drug-sensitive virus release. Antiviral analysis of OC found 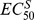 in the range of 0.0008 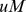 to >35 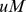 [21]. We tested our model assuming 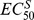 equalling 0.5, 5, 10 and 40 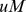 which correspond to an average efficacy at steady state of 0.999, 0.99, 0.98 and 0.93, respectively, for the standard therapeutic dose of 75 mg bid for 5 days.

#### Drug-sensitive virus dynamics

The time-course of influenza infection and symptoms were described by a model including target epithelial cells, infected cells, free virus, pro-inflammatory cytokines, NK cell activity, and systemic symptoms as shown in Fig. 1B [18]. The system of ODEs used is:
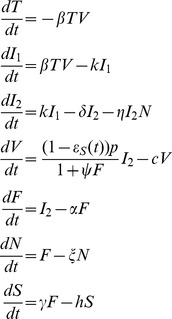
(2)where 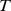 is the number of target cells, 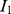 and 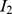 are the number of infected cells (non-productive and productive, respectively), 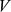 is the drug-sensitive virus titre, 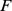 is the pro-inflammatory cytokine level, 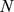 represents activated NK cells and 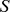 is the systemic symptom score, where 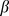 is the target cell infection rate, 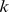 is the transition rate from 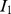 to 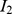, 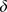 is the mortality rate of productively infected cells, 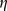 is the rate constant for NK cell killing of productively infected cells, 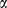 is the cytokine clearance rate, 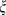 is the mortality rate of NK cells, 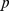 is the rate of virus production by 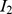, 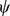 is a parameter describing the effect of cytokines on 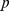, and 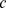 is the virus clearance rate. 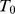, the initial number of target cells in the upper respiratory tract, was set to 4×10^8^ [22]. Finally, we assumed that a target cell can be infected by either drug-sensitive or drug-resistant virus, but not both, as was done previously [23].

Influenza viral titres were expressed in median tissue culture infective dose per millilitre (TCID_50_/mL) and systemic symptoms were expressed as a score representing the intensity of fever or feverish feeling, headache, muscle ache, and fatigue. The systemic symptom score ranged from 0 in the absence of systemic symptoms to 12 when all symptoms were at their highest intensity [18].

As we did not include the adaptive immune response in our model, we terminate simulations at day 8 after infection.

#### Drug-resistant virus dynamics

We modelled the emergence of drug-resistant virus as a random event: each virion produced by a cell infected with a drug-sensitive strain is resistant with probability 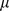. We assumed that the kinetics of resistant variant virus and wild-type virus were similar. Although resistance mutations, such as H275Y, can induce a loss of fitness, this loss can be restored by compensatory mutations [24], [25]. We thus explored the effect of both equal fitness and of a 10% fitness cost (reduction of the infectivity, β) for resistant virus compared to sensitive virus.

For large populations of resistant-strain infected cells and virus (above 10^4^) we model the resistant-strain dynamics with the ODEs
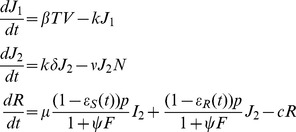
(3)where 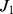 and 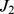 represent non-productive and productive resistant-strain infected cells, respectively, and 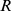 represents resistant virus. We modify the sensitive-strain dynamics accordingly, setting the target cell equation to 
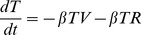
 and the sensitive-strain virus equation to 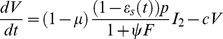. Note that we have included a drug efficacy against resistant virus 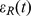, which is given by the same formula as 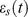 but with a different 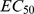; it has been shown that for the resistant virus with the mutation H275Y, the OC 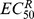 is 400 times higher than drug-sensitive virus 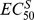 [26]. We also explored ratios of 200 to 800 in a sensitivity analysis.

As we are interested in the emergence of drug resistance, stochastic dynamics on small populations of resistant-strain infected cells and resistant virus are key. For small populations of these species, we use a stochastic approach. We employ a modified stochastic simulation algorithm (SSA) to model resistant virus dynamics; the propensity functions describing the viral kinetics of the resistant virus are shown in Table 1. The SSA was adapted to incorporate time-dependent oseltamivir pharmacokinetics (Text S1) and sensitive strain dynamics (Eq. 2), which we continue to model deterministically [27]. Only when the number of resistant viruses and infected cells grows beyond a fixed threshold, here set to 10^4^, do we transition from this hybrid deterministic/stochastic regime to a fully deterministic regime. We also set a lower threshold of 1, such that if the number of drug-sensitive infected cells and virus falls below this threshold, while resistant populations are at 0, we assume the infection cleared and stop the simulation.

**Table 1:** Stochastic model for resistant virus.

Event	propensity	State change (T,J_1_,J_2_,R)
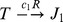	c_1_ = βTR	(−1,+1,0,0)
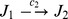	c_2_ = kJ_1_	(0,−1,+1,0)
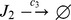	c_3_ = δJ_2_+τNJ_2_	(0,0,−1,0)
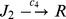	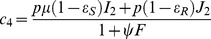	(0,0,0,+1)
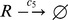	c_5_ = cR	(0,0,0,−1)

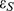
 and 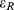 represent the effectiveness of oseltamivir on drug-sensitive and drug-resistant virus, respectively, with 
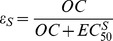
and 
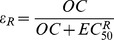
where OC is oseltamivir carboxylate concentration, 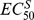 is the concentration necessary to observe 50% of the maximal effect on drug-sensitive virus and 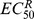 is the concentration necessary to observe 50% of the maximal effect on drug-resistant virus. 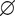 represents the absence of any element.

One challenge in using a stochastic approach is the issue of units. The model parameters were estimated to fit viral titer data expressed in TCID_50_/mL of nasal wash. With the stochastic component of our approach, we need to convert to the total number of infectious virions at the site of infection. Handel *et al.* [28] estimated a conversion factor between 10^2^ and 10^5^, which takes into consideration the volume of respiratory tract as well as the fact that there is probably more than one infectious virion in one TCID_50_ and that the virion concentration at the site of infection is probably larger than in nasal wash. We set our conversion factor to 1 TCID_50_/mL of nasal wash = 10^3^ virions at the site of infection. To implement this conversion factor, we rescaled the viral titer 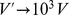, the virus production rate 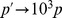 and the rate of infection 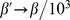. The prime distinguishes the corrected parameters that are used in the stochastic model.

To assess the effect of the correction we also tested a set of simulations for 75 mg bid for 5 days with the conversion factor set to 1 TCID_50_/mL = 10^2^ virions and 1 TCID_50_/mL = 10^4^ virions.

### Parameters

We simulated viral dynamics and investigated emergent drug resistance in a sample of 1000 individuals. The three individual PK parameters and eleven individual VKSD parameters were drawn from a log-normal distribution 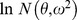, where 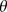 is the average population value and 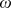 is the average between-individual variability [18], [20] (Text S2). 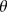 and 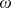 and their respective standard errors were estimated in previous studies for all parameters [18], [20]. These studies involved healthy volunteers who in the VKSD study were on 18 to 40 years old, with serum hemagglutinin antibody titers of <1∶8 to the relevant virus strains and experimentally infected with influenza A/Texas/91 at time 0.

Finally, the resistant variant emergence rate, 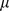, was set to 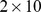 ^−6^ per replication cycle (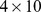 ^−6^ in the case of reduced infectivity of resistant strains) so that the model reproduces previous observations showing that between 0.4 and 1% of patients when treated with 75 mg bid for 5 days starting one day after symptom onset shed resistant virus 2 days after treatment initiation [29], [30].

### Drug regimens

Three oseltamivir regimens are approved in adults: 75 mg daily (qd) for 10 days (post-exposure prophylaxis regimen), 75 mg twice-a-day (bid) for 5 days (curative regimen) and 150 mg bid for 5 days (recommended regimen for severe pandemic influenza) [31]. In a first set of simulations, we used the recommended curative regimen as a reference. For comparison purposes, we explored the effect of drug dose simulating 75, 150 and 300 mg bid for 5 days. We also explored the effect of intake frequency, simulating 75 mg qd for 5 days, 75 mg bid for 5 days and 75 mg three-times-a-day (tid) for 5 days. Finally, we explored the effect of treatment duration, simulating 75 mg bid for 5, 10 and 15 days.

In a second set of simulations, we used the recommended post-exposure prophylaxis regimen as a reference. We investigated the effect of the dose simulating 75, 150 and 300 mg qd for 10 days. We also explored the effect of intake frequency, simulating 75 mg qd for 10 days, 75 mg bid for 10 days and 75 mg tid for 10 days. Finally, we examined the effect of prophylaxis duration, simulating 75 mg qd for 5, 10 and 15 days.

We chose ten possible therapy initiation times. Therapy initiation was simulated at 3, 2 and 1 day(s) prior to infection in order to study the impact of possible drug build-up before infection occurred. To simulate therapy initiation during the incubation period, the time of first intake was set to 0, 0.5, 1 and 1.5 days after infection. To simulate a typical curative regimen, we set the first intake at 2, 3 and 4 days after infection, which corresponded to 0, 1 and 2 days after symptom onset on average [6]. In all cases, the VKSD was simulated from the time of infection until day 8.

Finally, to simulate imperfect adherence, we considered an early discontinuation of therapy after 4 and 6 intakes (instead of 10) for 75 mg qd and 75 mg bid regimens.

We call therapies initiated after symptom onset treatment and those started before symptom onset prophylaxis.

### Endpoints

We assessed primarily the virological efficacy, defined here as the average decrease of the area under the curve of drug-sensitive virus titer (without transformation) (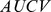) and drug-resistant virus titer (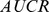) under treatment relative to the AUC of viral titers without antiviral therapy (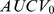 and 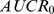). Virological efficacy was computed as:
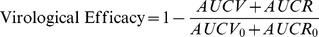



Symptom efficacy was measured as the average decrease in AUC of systemic symptoms (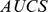) under antiviral therapy relative to the AUCS without antiviral therapy (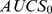). Symptom efficacy was thus computed as:
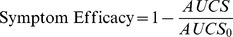



The results are presented as the median value and inter-quartile range (IQR). Two criteria were used to measured resistance emergence: first, the proportion of patients shedding resistant virus above the limit of detection (LOD = 2 TCID_50_/mL [32]). We also assessed the fraction of all virus shed that was resistant. All results about viral shedding presented thereafter represent viral load (for drug-sensitive and/or drug-resistant virus) above the LOD, at any time of infection.

## Results

### Pharmacokinetics of antiviral regimens

**Figure 2:**
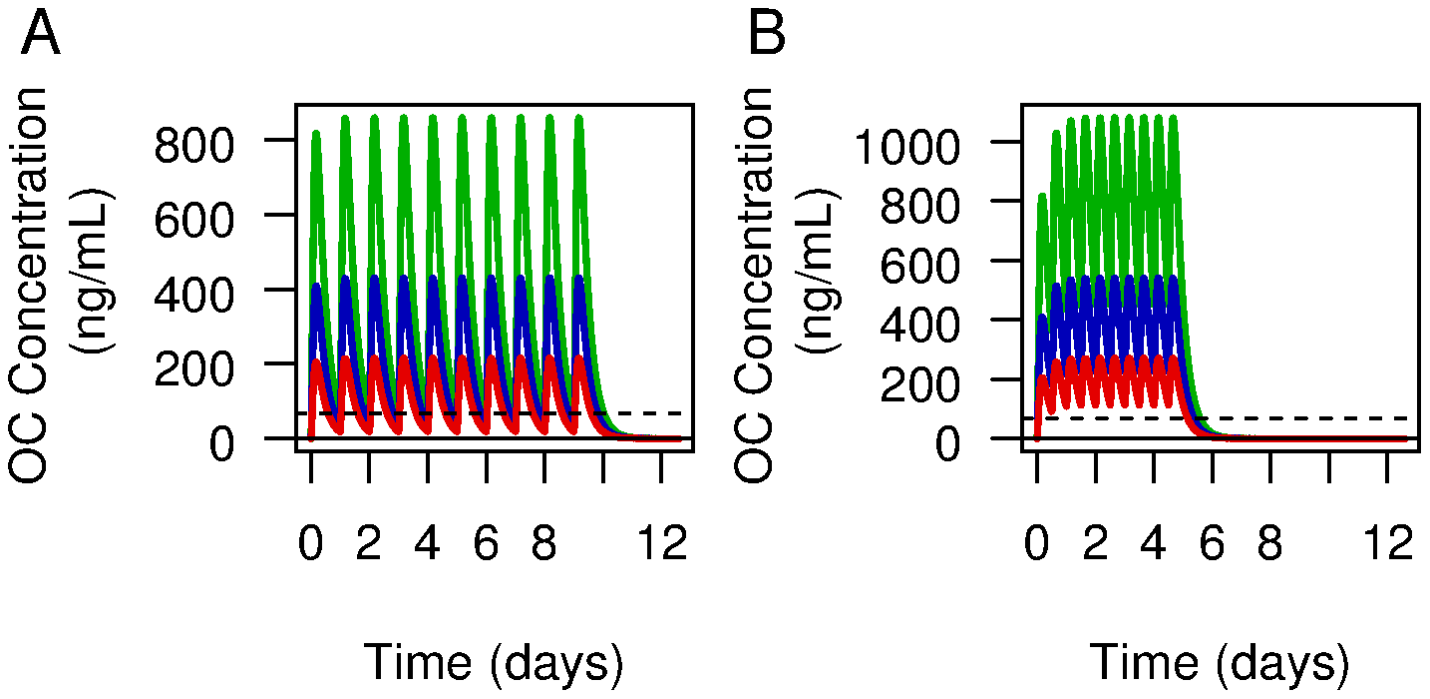
Average oseltamivir carboxylate kinetics (red line: 75 mg, blue line: 150 mg, green: 300 mg). Panel A shows the pharmacokinetics for twice-a-day intake for 5 days and the panel B once a day intake for 10 days. The black line represents the 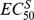 for drug-sensitive virus and the dashed line the 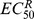 for resistant virus. The 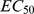
s were converted from 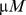 to ng/mL for this figure.

Our simulated PK model shows increases of OC concentration with peaks occurring on average 4 hours after each intake (Fig. 2.). After the last oseltamivir intake, a prolonged concentration decrease was observed (Fig. 2). The height of the peak was proportional to the regimen dose. OC concentrations above the 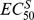 for drug-sensitive and resistant viruses were obtained in the first hour after first oseltamivir intake, regardless of dose. While the OC concentration remained above the 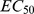 for both drug-sensitive and drug-resistant viruses with twice-a-day intakes, with 75 mg qd for 10 days, OC concentration was above the 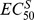 for drug-sensitive virus and below the 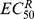 for resistant virus for an average 7.8 hr after each intake.

### Viral kinetics, symptom dynamics and resistance without antiviral therapy

**Figure 3:**
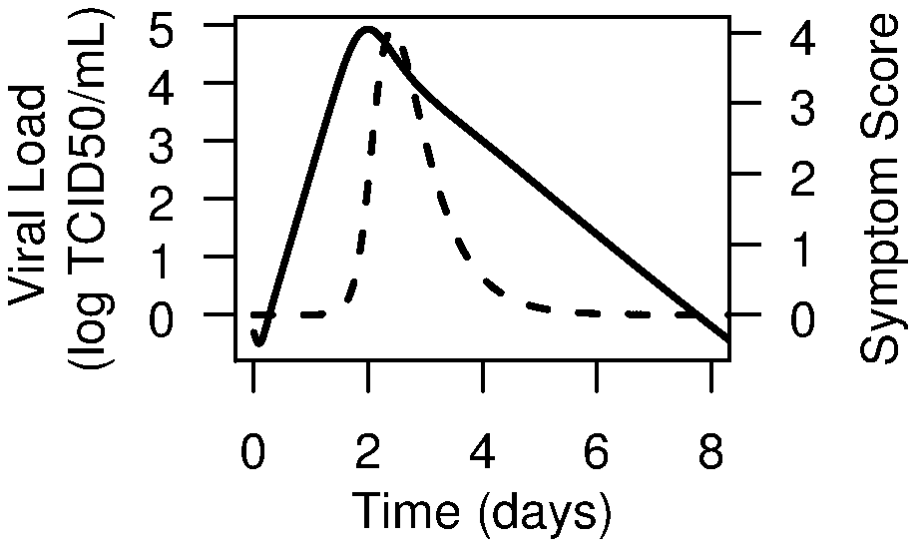
Average viral kinetics and symptom dynamics. The solid line represents influenza viral kinetics and the dashed line the systemic symptom dynamics without antiviral therapy.

Simulating 1000 *in silico* patients, drawing parameters from previously estimated distributions [18], [20], we find the median viral titer peak was 4.5 log_10_ (TCID_50_/mL) (IQR 2.6–5.3 log_10_(TCID_50_/mL) (Fig. 3). The duration of viral shedding above the LOD was 7.0 days (IQR 1.0–8.0 days). The incubation period was 1.9 days (IQR 1.0–3.0) and the systemic symptom score peak was 3.9 (IQR: 0.1–12) and lasted 2.4 days (IQR 0.2–7.0 days). The AUC of the viral curve was 4.5 log_10_ (TCID_50_/mL) (IQR 2.9–5.3). The AUC of the systemic symptom curve was 2.0 (IQR 0.3–7.7). As expected due to our choice of μ, 0.6% patients shed resistant viruses.

### Virological and symptom efficacies of antiviral regimens

**Figure 4:**
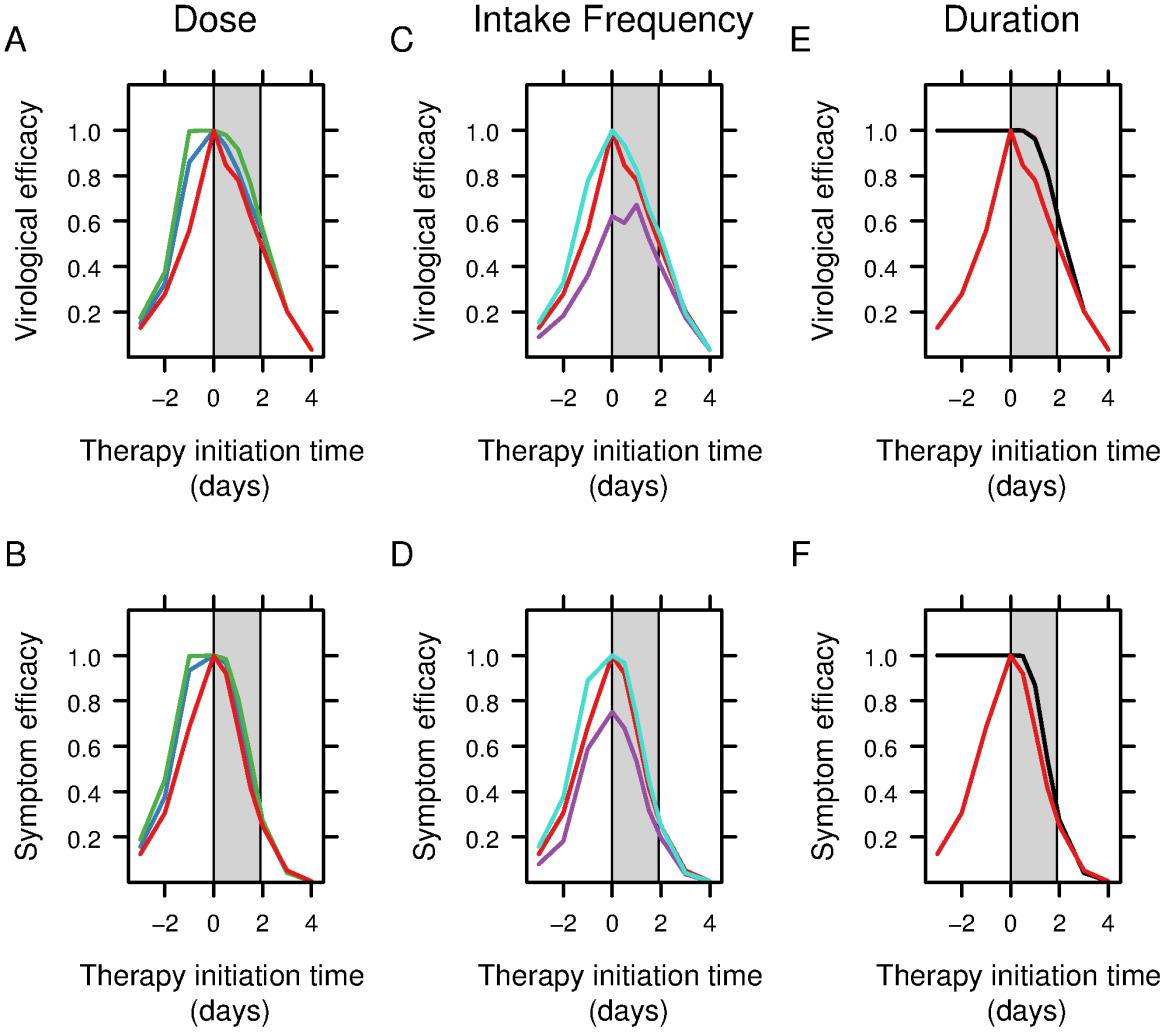
Oseltamivir efficacy measured in terms of virological and symptom efficacy. Each panel represents the variation of efficacy depending on the therapy initiation time relative to the time of infection. 0 indicates the time of inoculation and the grey rectangle the incubation period. Comparison of the effect of three possible doses (red: 75 mg bid for 5 days, blue: 150 mg bid 5 days, green: 300 mg bid 5 days) used to treat influenza on (A) virological efficacy and (B) symptom efficacy; Comparison of the effect on (C) virological efficacy and (D) symptom efficacy of three intake frequency (red: 75 mg bid for 5 days, purple: 75 mg qd for 5 days, turquoise: 75 mg tid for 5 days) used to treat influenza; Comparison of the effect on (E) virological efficacy and (F) symptom efficacy of three therapy durations (red: 75 mg bid for 5 days, pink: 75 mg bid for 10 days, black: 75 mg bid for 15 days) used to treat influenza.

Using the same set of 1000 *in silico* patients, but now given treatment we find that the virological and symptom efficacies (see Methods) were strongly dependent on the therapy initiation time (Fig. 4). For all possible drug regimens, both virological and symptom efficacies were found to be greatest when prophylaxis was initiated between one day before and one day after inoculation. With the recommended curative regimen of 75 mg bid for 5 days (red curve in all panels), virological efficacy during this period was above 60.0% with a peak at 99.9% for therapy initiated at the time of inoculation. When oseltamivir (75 mg bid for 5 days) was started earlier, the efficacy decreased as viral replication was postponed after the last intake, as shown by the high number of infected cells in many patients after the last intake and later (Fig. S1). The virological efficacy decreased with treatments started after symptom onset and is below 5.0% when the treatment is initiated at day 4 (Fig. 4). This low virological efficacy is due to the fact that the viral infection is largely resolved by this time in the absence of treatment (Fig. 3) A similar pattern was observed for symptom efficacy with a peak of 99.9% with 75 mg bid for 5 days initiated at the time of inoculation (Fig. 4B, D, F).

We next compared the effect of the dose on the variation of virological and symptom efficacy depending on the therapy initiation time relative to the time of infection (Figs. 4 A & B). The virological and symptom efficacies increased with higher doses when the oseltamivir is initiated before inoculation and were similar for treatments started after symptom onset (Table 2, Figs. 4 A & B). For example, for oseltamivir started 1 day after inoculation, the virological efficacy was 79.6% with 75 mg bid for 5 days, 83.7% with 150 mg bid for 5 days and 92.5% with 300 mg bid for 5 days (Table 2).

**Table 2:** Median virological efficacy for different therapy initiation times and drug regimens.

		Therapy initiation time (days)			
Variable tested	Regimen	−1	0.5	2	4
Dose	75 mg bid, 5 days*	0.615 (0.093–0.999)	0.863 (0.433–0.999)	0.554 (0.155–0.863)	0.036 (0.001–0.502)
	150 mg bid, 5 days*	0.870 (0.156–0.999)	0.969 (0.496–0.999)	0.597 (0.156–0.908)	0.036 (0.001–0.510)
	300 mg bid, 5 days	0.997 (0.315–0.999)	0.985 (0.563–0.999)	0.617 (0.158–0.933)	0.036 (0.001–0.518)
Intake frequency	75 mg qd, 5 days	0.441 (0.57–0.990)	0.640 (0.270–0.997)	0.473 (0.141–0.808)	0.036 (0.001–0.489)
	75 mg bid, 5 days*	0.615 (0.093–0.999)	0.863 (0.433–0.999)	0.554 (0.155–0.863)	0.036 (0.001–0.502)
	75 mg tid, 5 days	0.807 (0.152–0.999)	0.941 (0.515–0.999)	0.584 (0.157–0.898)	0.036 (0.001–0.511)
Treatment duration	75 mg bid, 5 days*	0.615 (0.093–0.999)	0.863 (0.433–0.999)	0.554 (0.155–0.863)	0.036 (0.001–0.502)
	75 mg bid, 10 days	0.999 (0.999–1.0)	0.999 (0.993–0.999)	0.639 (0.167–0.924)	0.036 (0.001–0.509)
	75 mg bid, 15 days	0.999 (0.999–1.0)	0.999 (0.994–0.999)	0.639 (0.167–0.924)	0.036 (0.001–0.509)
Pre-exposure prophylaxis	75 mg qd, 10 days*	0.999 (0.999–0.999)	0.999 (0.968–0.999)	0.619 (0.167–0.869)	0.036 (0.001–0.498)

The inter-quartile range (IQR) is given between brackets.

* currently approved regimens.

We then compared the effect of intake frequency on the variation of virological and symptom efficacy depending on the therapy initiation time relative to the time of infection (Figs, 4 C & D). The virological and symptom efficacies increased with higher intake frequency for prophylaxis and curative treatments (Table 2, Figs. 4C & D). For example, for oseltamivir initiated 1 day after inoculation, the virological efficacy was 68.6% with 75 mg qd for 5 days, 79.6% with 75 mg bid for 5 days and 85.1% with 75 mg tid for 5 days (Table 2).

We finally compared the effect of therapy duration on the variation of virological and symptom efficacy depending on the therapy initiation time relative to the time of infection (Figs. 4 E & F). The virological and symptom efficacies were greater for oseltamivir given for 10 days or more compared to therapy lasting 5 days (Table 2, Figs. 4 E & F). For example, for oseltamivir started 1 day after inoculation, the virological efficacy was 79.6% with 75 mg bid for 5 days, 97.0% with 75 mg bid for 10 days and 97.0% with 75 mg bid for 15 days (Table 2).

The difference in virological and symptom efficacy depending on the dose, therapy duration and intake frequency was below 1% (Table 2, Fig. 4) for treatment initiated 3 or 4 days after inoculation (or 1 or 2 days after symptoms onset).

For the set of simulation using the recommended post-exposure prophylaxis regimen (75 mg qd for 10 days) as reference, both virological and symptom efficacy remained high (above 99.9%) when the prophylaxis was administered for more than 10 days and when initiation took place before inoculation or 0.5 days after inoculation. Virological efficacy fell to 62% for a prophylaxis initiated 1 day after inoculation and below 5.0% when the curative treatment was initiated 4 days after inoculation (Fig. S2).

### Resistance emergence

Resistance emergence can occur before therapy initiation but the median fraction of resistant virus represented less than 0.001% of the total amount of virus shed. Without treatment or before therapy initiation, the viral titer for drug-resistant virus followed the same pattern as the drug-sensitive virus and peaked at the exact same time as we have assumed no fitness cost associated with resistance, i.e., the viral dynamic parameters are the same as for the drug-sensitive virus. Once under therapy, the drug-sensitive virus titer decreased rapidly while in patients shedding resistant virus the resistant virus titer increased (Fig. S4).

**Figure 5:**
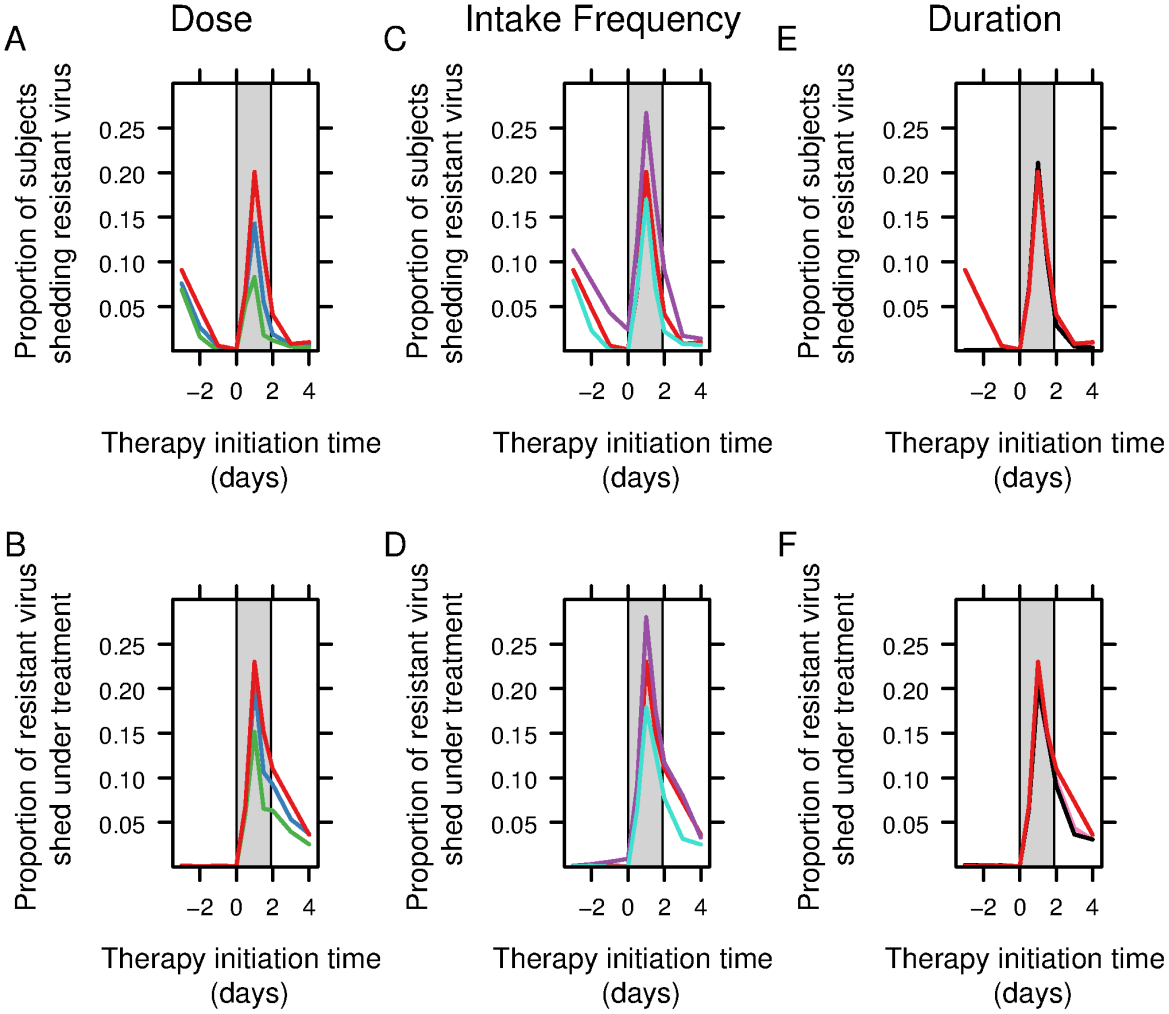
Resistance emergence measured in terms of the proportion of subjects shedding resistant virus and the fraction of resistant virus shed. Each panel represents the variation of resistance emergence depending on the therapy initiation time relative to the time of infection. 0 indicates the time of inoculation and the grey rectangle the incubation period. Comparison of the effect on (A) the proportion of subjects shedding resistant virus and (B) the fraction of resistant virus shed of three possible doses (red: 75 mg bid for 5 days, blue: 150 mg bid 5 days, green: 300 mg bid 5 days) used to treat influenza; Comparison of the effect on (C) the proportion of subjects shedding resistant virus and (D) the fraction of resistant virus shed of three intake frequency (red: 75 mg bid for 5 days, purple: 75 mg qd for 5 days, turquoise: 75 mg tid for 5 days) used to treat influenza. Comparison of the effect on (E) the proportion of subjects shedding resistant virus and (F) the fraction of resistant virus shed of three therapy durations (red: 75 mg bid for 5 days, pink: 75 mg bid for 10 days, black: 75 mg bid for 15 days) used to treat influenza.

We studied the variation of resistance emergence depending on the therapy initiation time relative to the time of infection for a set of different regimens. We observed a similar pattern of resistance emergence with the different regimens (Figs. 5 and S3).

If a 5-day prophylaxis regimen was initiated before inoculation, the proportion of subjects shedding resistant virus increased the earlier prophylaxis was initiated. This reflects the fact that drug concentration above 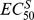 was not available long enough to suppress viral replication. This was observed for every simulated regimen except when prophylaxis lasted 10 or 15 days. In these latter cases, the proportion of subjects shedding resistant virus when the prophylaxis was initiated before inoculation remained below 1%. The fraction of resistant virus shed remained below 1% when prophylaxis was initiated before inoculation except for the recommended post-exposure prophylaxis regimen (75 mg qd for 10 days) for which it was between 1.9 and 2.5% (Fig. S3).

The proportion of subjects shedding resistant virus increased when oseltamivir was initiated during the incubation period with a peak between 8.3% (300 mg bid for 5 days) and 26.7% (75 mg qd for 5 or 10 days) for therapy initiated 1 day after inoculation (Figs. 5 and S3). Similarly the fraction of drug-resistant virus peaked for therapy initiated 1 day after inoculation. Our model predicts a peak in resistance emergence for any regimen when the first oseltamivir intake takes place 1 day after inoculation (Fig. 5 A–F). The proportion of resistant virus then decreased with the time treatment was administered after symptom onset.

Looking in more detail at the effect of dose on resistance emergence depending on the therapy initiation time we note resistance emergence decreased with higher doses when oseltamivir was initiated before inoculation and was similar for treatments started after symptom onset (Figs. 5 A & B). For example, for oseltamivir started 1 day after inoculation, the proportion of subjects shedding resistant virus was 20.1%, 14.3% and 8.3% with 75 mg, 150 mg and 300 mg bid for 5 days, respectively.

We then compared the effect of the intake frequency on resistance emergence depending on the therapy initiation time relative to the time of infection (Figs. 5 C & D). Resistance emergence decreased with higher intake frequency for prophylaxis and curative treatments (Figs. 5C & D). For example, for oseltamivir started 1 day after inoculation, the proportion of subjects shedding resistant virus was 26.7%, 20.1% and 17.0% with 75 mg given for 5 days qd, bid and tid, respectively.

We finally compared the effect of therapy duration on resistance emergence. Resistance emergence was similar with 75 mg bid for 5, 10 and 15 days (Figs. 5E & F). For example, for a oseltamivir started 1 day after inoculation, the proportion of subject shedding resistant virus was 20.1%, 21.0% and 21.0% with 75 mg bid for 5, 10 and 15 days, respectively.

### Early therapy discontinuation

As expected, early therapy discontinuation was associated with a decrease of virological and symptom efficacies and with an increase of the proportion of subjects shedding resistant virus. When therapy with 75 mg bid started 1 day after inoculation the virological efficacy decreased from 79.6% for a 5 day therapy to 49.0% for a discontinuation after 4 intakes and 67.5% for a discontinuation after 6 intakes; symptom efficacy decreased from 72.3% to 44.5% and 46.3% for discontinuations after 4 and 6 intakes, respectively. The proportion of subjects shedding resistant virus increased from 20.1% to 26.4% and 22.6%, for discontinuations after 4 and 6 intakes, respectively (Fig. S7).

### Sensitivity analyses

We simulated viral kinetics under oseltamivir using four different 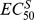 to reflect the wide range of values experimentally measured in antiviral analysis [21]. The pattern was similar with all four 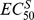, with maximal efficacy obtained when therapy was initiated between 1 day before and 1 day after inoculation and a peak of resistance emergence when oseltamivir was initiated during the incubation period. As expected, we found that virological and symptom efficacy would decrease, while resistance emergence would increase, when the 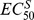 was increased (Fig. S5). Drug efficacy varied inversely with 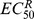 and resistance emergence increased with 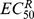 (Fig. S6 E–H).

When we tested the effect of the conversion factor from TCID_50_/mL to the number of infectious virions at the site of infection, we found similar results. The virological and symptom efficacies dependency on oseltamivir initiation time was similar for all conversion factors. However, we noted an impact on the stochastic simulations as the variability of drug-resistant virus shedding is larger for smaller conversion factors. This led to similar virological and symptom efficacies but resistance emergence increased when the conversion factor was decreased (Fig. S5) as more subjects shed drug-resistant virus above the LOD (Fig. S6).

An infectivity decrease by 10% for resistant virus had a limited impact on the proportion of subjects shedding resistant virus above the limit of detection and the proportion of virus shed that is resistant (Fig. S6 A–D). Similarly, the curves showing how the virological and symptom efficacies vary with treatment initiation time were similar to those found in the absence of any cost due to resistance (Fig. S6).

## Discussion

Mathematical modeling has provided significant insights into influenza viral kinetics [22], [23], [33], [34], [35], [36], [37].

Handel et al. used a viral kinetic model for characterizing emergence of drug resistance during oseltamivir treatment [28], but they did not consider the effects of drug PK nor did they take into account the between-individual variability. Using the population approach, our model reflects the heterogeneity in viral kinetic and symptoms dynamic observed in the population. This allowed us to compute the proportion of subjects shedding resistant virus and therefore to assess resistance emergence.

In this previous modeling study, efficacy was considered constant over time and ineffective on drug-resistant strains [28]. In contract, we allowed the drug-resistant strain to be sensitive to high OC concentrations [26]. Moreover, in our model the drug efficacy can vary greatly due to drug concentration fluctuations and between-subject variability with respect to both their viral kinetics and pharmacokinetics. We found that the OC concentration with the recommended prophylaxis regimen of 75 mg qd was above the 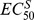 for drug-sensitive virus and below the 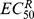 for drug-resistant virus for an average of 7.8 hr between two doses. These low concentrations favor the selection of resistant virus.

Our approach using a hybrid stochastic and deterministic simulation algorithm is capable of capturing the stochastic behavior of small populations of viruses and infected cells while reducing the computational burden associated with fully stochastic algorithms. The partitioned leaping algorithm [38] employed by Handel *et al.* [28] to investigate a related problem in emergent neuraminidase inhibitor resistance is also well suited to problems with widely disparate rates and species populations. This algorithm assumes constant rate parameters. However, as we incorporated oseltamivir pharmacokinetics into our model, yielding time-dependent drug efficacies, which translate in our simulation to time-dependent rates, the partitioned leaping algorithm does not lend itself well to simulations of our model and was not pursued. Instead the hybrid stochastic and deterministic simulation algorithm was used as it is well adapted to models with time-dependent rates– as is the case in the present study, where we incorporated both viral dynamics and innate immune responses. Using this approach, we were able to perform model simulations under a wide variety of drug regimens and investigated further the effect of numerous covariates such as treatment initiation time, dose, intake frequency and treatment duration on efficacy and resistance emergence risk.

In our model, we assumed that every subject was infected with drug-sensitive virus, which is the most optimistic scenario, and we did not consider cases in which subjects were infected with resistant virus or a mixture of both. If we allowed infection by resistant virus the selection of resistant virus would occur faster and the proportion of subjects shedding resistant virus and the fraction of resistant virus would dramatically increase.

We also assumed that only resistant virus with the same fitness as drug-sensitive virus emerged. Pre-existing permissive mutations, such as R222Q, R194G, E214D, T289M, N369K, L250P or F294Y, in the drug-sensitive virus were shown to facilitate the emergence of resistant virus without any fitness loss [25], [39], [40]. In case of a mixture of resistant virus (H275Y) with and without a permissive mutation [41], the virus with the permissive mutation grows faster than the virus without this mutation, and therefore the amount of resistant virus with fitness loss would be negligible.

Our simulations show an increased efficacy for early oseltamivir initiation as the drug blocks virus release and subsequent rounds of virus infection. In case of oseltamivir initiation before infection, we show that efficacy decreases for short duration of treatment as infected cells are not cleared at the end of treatment. Efficacies are also low in case of late initiation (after symptom onset) of oseltamivir as the infection is already resolving. The peak in the amount of resistant virus shed and in the proportion of people shedding resistant virus observed when oseltamivir is initiated during the incubation period (Fig. 5) can be explained by the selective pressure of oseltamivir on drug-sensitive strains and by the fact that at the early stage of infection there is a lack of sufficient cytokines to control the resistant virus and to stimulate the NK cell response.

Our simulations were consistent with previous results in terms of virological and symptom efficacies: early administration of oseltamivir increased virological and symptom efficacy [42], [43], [44]. More specifically, the mean virological efficacy with 75 mg bid was 86.3% when oseltamivir was taken 0.5 days after inoculation and fell to 24% when oseltamivir was taken 3 days after inoculation, which is similar to published experimental and epidemiologic data [3], [45].

The rates of emerging resistance predicted by our model are consistent with previously described experimental and epidemiological studies [16], [46], [47], [48], irrespective of whether the first intake was 1 day prior to inoculation, during the incubation period or after symptom onset.

We found that the timing of therapy initiation is crucial to reach the right balance between efficacy and resistance emergence as it provides a global pattern that dose, intake frequency and treatment duration can modulate.

Resistance emergence dramatically increased when prophylaxis was initiated during the incubation period. This was observed for every dose regimen and was exacerbated with low dose or when oseltamivir was taken once-a-day. For instance, with the recommended post-exposure prophylactic regimen (75 mg qd for 10 days), the proportion of subjects shedding resistant virus increased from 2.2% when prophylaxis was initiated before inoculation to 26.7% when prophylaxis was initiated during the incubation period and fell below 1% when treatment was initiated after symptom onset. Whereas the proportion of subjects shedding resistant virus was below 1% with treatment initiated after symptom onset, it dramatically increased and reached up to 27% with prophylaxis initiated during the incubation period. Similarly, expected the fraction of resistant virus shed by an infected person rose to 23% with prophylaxis initiated during the incubation period.

We explored the effect of imperfect adherence on resistance emergence and drug efficacy (Fig. S7). We found that early discontinuation of treatment induces a decrease of both virological and symptom efficacies and an increase of the risk of resistance emergence.

Our model also predicts that it is very unlikely that an individual on long-term prophylaxis (75 mg qd for 10 or 15 days) will become infected by a drug-sensitive strain (Fig. S2), in line with epidemiologic findings [49], [50]. However, this probability increases dramatically in case of an infection with a resistant virus (Text S3). Consequently, prophylaxis failure is likely caused either by infection with a resistant virus or by selection of a *de novo* resistant virus and switching to a curative regimen in those patients would have no impact on the time course of influenza.

Our findings have several limitations. First, this study is limited to otherwise healthy adult subjects infected with drug-sensitive virus only, as parameters were obtained from studies conducted in this setting [18]. In other populations, such as children the pharmacokinetics might substantially differ. Indeed, the recommended oseltamivir dose is 1.0 mg/kg and 2.0 mg/kg bid in neonates and children aged 1–5 years, respectively, leading to lower concentrations than in adults [14], [51], [52]. This in part could explain the increased risk of emerging resistance observed in children [14], [53], [54].

Second, our model did not include an adaptive immune response. In the study subjects from which our viral kinetic parameters were derived, the anti-influenza antibody titer was below 1∶8, suggesting that they did not have prior exposure to this strain of influenza. Thus our predictions might not apply to a population with high-level pre-existing immunity. In future studies one could use a model such as that of Miao et al. [37] or others [23], [34], [35], [36] that include adaptive immune responses. However, such models have not been well parameterized for human infections, especially with regard to individual variability.

Third, our model was based on experimental infection studies where the time of infection was known. Thus, we could study the effect of starting treatment at defined times relative to the time of infection. For naturally acquired infections, the time of infection is not known although if severe enough the time of symptom onset can be identified. Our model suggests that starting therapy during the incubation period, i.e. pre-symptoms, can enhance the probability of drug resistance emerging during therapy. Starting prophylaxis during the incubation period may occur in a household setting where an index case is diagnosed with influenza infection and his/her asymptomatic contacts start oseltamivir therapy to prevent new cases.

In summary, we found that the recommended post-exposure prophylactic regimen should be used with caution, as it increases the risk of emerging resistance [55]. More specifically, once-a-day intake increases the proportion of subjects shedding resistant virus by 2 to 6% across all simulated dose regimes. Most importantly, initiating prophylaxis during the incubation period is the main factor leading to resistance emergence for all possible regimens. During this period, the infected cells cannot be controlled by NK cells as many of them are not yet activated and the number of target cells is still large. We thus believe that oseltamivir prophylaxis should be restricted either to subjects prone to develop severe cases (such as immunocompromised subjects) and treated with high doses (300 mg per intake), frequent intakes (bid or tid) and for longer duration (10 to 15 days), or in the otherwise healthy patients, after exclusion of an influenza infection in the incubation period (for example using a sensitive rapid test), in order to decrease the risk of resistant virus emergence and to preserve oseltamivir efficacy.

## Supporting Information

Figure S1 **Evolution of the number of infected cells with a treatment of 75 mg bid started 2 days before inoculation and without interruption date.** 15 days after inoculation, the infected cells were cleared in 93% of patients.
(DOCX)

Figure S2 **Oseltamivir efficacy.** Each panel represents the variation of efficacy depending on the therapy initiation time relative to the time of infection. 0 stands for the time of inoculation and the grey rectangle the incubation period. Comparison of the effect on (A) virological efficacy and (B) on symptom efficacy of three possible doses (red: 75 mg qd for 10 days, blue: 150 mg qd 10 days, green: 300 mg qd 10 days) used to treat influenza; Comparison of the effect on (C) virological efficacy and (D) on symptom efficacy of three intake frequency (red: 75 mg qd for 10 days, purple: 75 mg bid for 10 days, turquoise: 75 mg tid for 10 days) used to treat influenza; Comparison of the effect on (E) virological efficacy and (F) on symptom efficacy of three therapy durations (red: 75 mg qd for 10 days, pink: 75 mg qd for 15 days, black: 75 mg qd for 5 days)used to treat influenza.
(DOCX)

Figure S3 **Resistance emergence.** Each panel represents the variation of resistance emergence depending on the therapy initiation time relative to the time of infection. 0 stands for the time of inoculation. 0 stands for the time at inoculation and the grey rectangle the incubation period. Comparison of the effect on (A) the proportion of subjects shedding resistant virus and (B) on the proportion of resistant virus shed under of three possible doses (red: 75 mg qd for 10 days, blue: 150 mg qd 10 days, green: 300 mg qd 10 days) used to treat influenza; Comparison of the effect on (C) the proportion of subjects shedding resistant virus and (D) on the proportion of resistant virus shed under of three intake frequency (red: 75 mg qd for 10 days, purple: 75 mg bid for 10 days, orange: 75 mg tid for 10 days) used to treat influenza; Comparison of the effect on (E) the proportion of subjects shedding resistant virus and (F) on the proportion of resistant virus shed under of three therapy durations (red: 75 mg qd for 10 days, pink: 75 mg qd for 15 days, brown: 75 mg qd for 5 days) used to treat influenza.
(DOCX)

Figure S4 **A) Individual viral load (drug-sensitive+drug resistant virus) B) Individual ratio of resistant virus to total virus shed depending on time after infection.** (y-axis is in log scale) In red, patients with resistant virus emerging and in grey patients without resistant virus emerging. Sample of 100 subjects.
(DOCX)

Figure S5
**Sensitivity analysis.** Comparison, for subjects under 75 mg bid of oseltamivir for 5 days, of the effect of the correction term (panels A to D) for 10^2^ (red), 10^3^ (green) and 10^4^ (blue). Note that we used the mutation rate defined previously (2 10^6^) and comparison of the effect of 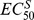 for the resistant virus (panels E to H) for 0.5 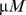
(red), 5 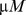 (blue) and 10 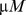 (green). Note that we used the mutation rate defined previously (2 10^6^). We also considered in all cases that 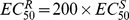.(DOCX)

Figure S6
**Sensitivity analysis 2.** Comparison of the effect of the infectivity cost (panels A to D) for 10% (blue) and no infectivity cost (red) and comparison of the effect of 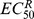 for the resistant virus (panels E to H) for 200 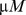 (green), 400 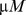 (red) and 800 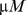 (blue).(DOCX)

Figure S7 **Imperfect adherence.** Comparison of the effect of early treatment termination for 75 mg bid (panels A to D) after 10 intakes (red), 6 intakes (green), 4 intakes (blue) and comparison of the effect of early treatment termination for 75 mg qd (panels E to H) after 10 intakes (red), 6 intakes (green) and 4 intakes (blue).
(DOCX)

Figure S8 **Effect of conversion factor on individual drug-resistant virus viral shedding.** (A) 1 TCID_50_/mL = 10^2^ virions; (B) 1 TCID_50_/mL = 10^3^ virions; (C) 1 TCID_50_/mL = 10^4^ virions; The dashed line represents the limit of detection (LOD) of influenza virus.
(DOCX)

Text S1 **PK model: Analytical solution.**
(DOCX)

Text S2 **Population approach.**
(DOCX)

Text S3 **Probability of extinction.**
(DOCX)
