# Correction: Impact of Different Oseltamivir Regimens on Treating Influenza A Virus Infection and Resistance Emergence: Insights from a Modelling Study

**DOI:** 10.1371/journal.pcbi.1003699

**Published:** 2014-06-03

**Authors:** The PLOS Computational Biology Staff

There are two corrections to this manuscript:

The legend for Figure 2 is incorrect. The complete, correct legend for Figure 2 is:

**Figure 2. Average oseltamivir carboxylate kinetics (red line: 75 mg, blue line: 150 mg, green: 300 mg).** Panel A shows the pharmacokinetics for once a day intake for 10 days and the panel B twice-a-day intake for 5 days. The black line represents the 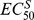 for drug-sensitive virus and the dashed line the 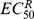 for resistant virus. The 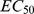
s were converted from 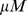
to ng/mL for this figure.

The first sentence of the final paragraph of the Discussion section should have cited reference 48 instead of reference 55.

The correct sentence should read: In summary, we found that the recommended post-exposure prophylactic regimen should be used with caution, as it increases the risk of emerging resistance [48].

Reference 48: Fry AM, Goswami D, Nahar K, Sharmin AT, Rahman M, et al. (2014) Efficacy of oseltamivir treatment started within 5 days of symptom onset to reduce influenza illness duration and virus shedding in an urban setting in Bangladesh: a randomised placebo-controlled trial. Lancet Infect Dis 14: 109–118. doi: 10.1016/s1473-3099(13)70267-6
