# Supplementary material for: Impact of Different Oseltamivir Regimens on Treating Influenza A Virus Infection and Resistance Emergence: Insights from a Modelling Study
Source: PLoS Comput Biol. 2014 Apr 17;10(4):e1003568. doi: 10.1371/journal.pcbi.1003568 (PMC3990489; doi:10.1371/journal.pcbi.1003568)
Supplement: Figure S1 — Evolution of the number of infected cells with a treatment of 75 mg bid started 2 days before inoculation and without interruption date. 15 days after inoculation, the infected cells were cleared in 93% of patients. (DOCX) [file pcbi.1003568.s001.docx]

**Supporting information**


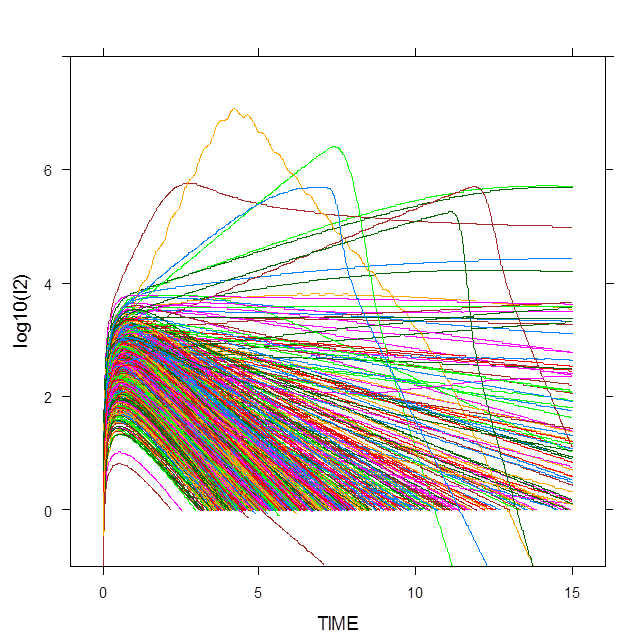


**Figure S1:** Evolution of the number of infected cells with a treatment of 75mg bid started 2 days before inoculation and without interruption date. 15 days after inoculation, the infected cells were cleared in 93% of patients.
