# Supplementary material for: Impact of Different Oseltamivir Regimens on Treating Influenza A Virus Infection and Resistance Emergence: Insights from a Modelling Study
Source: PLoS Comput Biol. 2014 Apr 17;10(4):e1003568. doi: 10.1371/journal.pcbi.1003568 (PMC3990489; doi:10.1371/journal.pcbi.1003568)
Supplement: Figure S3 — Resistance emergence. Each panel represents the variation of resistance emergence depending on the therapy initiation time relative to the time of infection. 0 stands for the time of inoculation. 0 stands for the time at inoculation and the grey rectangle the incubation period. Comparison of the effect on (A) the proportion of subjects shedding resistant virus and (B) on the proportion of resistant virus shed under of three possible doses (red: 75 mg qd for 10 days, blue: 150 mg qd 10 days, green: 300 mg qd 10 days) used to treat influenza; Comparison of the effect on (C) the proportion of subjects shedding resistant virus and (D) on the proportion of resistant virus shed under of three intake frequency (red: 75 mg qd for 10 days, purple: 75 mg bid for 10 days, orange: 75 mg tid for 10 days) used to treat influenza; Comparison of the effect on (E) the proportion of subjects shedding resistant virus and (F) on the proportion of resistant virus shed under of three therapy durations (red: 75 mg qd for 10 days, pink: 75 mg qd for 15 days, brown: 75 mg qd for 5 days) used to treat influenza. (DOCX) [file pcbi.1003568.s003.docx]

**Supplementary information**

**Figure S3: Resistance emergence**: Each panel represents the variation of resistance emergence depending on the therapy initiation time relative to the time of infection. 0 stands for the time of inoculation. 0 stands for the time at inoculation and the grey rectangle the incubation period.

Comparison of the effect on (A) the proportion of subjects shedding resistant virus and (B) on the proportion of resistant virus shed under of three possible doses (red: 75mg qd for 10 days, blue: 150 mg qd 10 days, green: 300mg qd 10 days) used to treat influenza; Comparison of the effect on (C) the proportion of subjects shedding resistant virus and (D) on the proportion of resistant virus shed under of three intake frequency (red: 75 mg qd for 10 days, purple: 75mg bid for 10 days, orange: 75mg tid for 10 days) used to treat influenza; Comparison of the effect on (E) the proportion of subjects shedding resistant virus and (F) on the proportion of resistant virus shed under of three therapy durations (red: 75 mg qd for 10 days, pink: 75mg qd for 15 days, brown: 75mg qd for 5 days) used to treat influenza.
