# Supplementary material for: Impact of Different Oseltamivir Regimens on Treating Influenza A Virus Infection and Resistance Emergence: Insights from a Modelling Study
Source: PLoS Comput Biol. 2014 Apr 17;10(4):e1003568. doi: 10.1371/journal.pcbi.1003568 (PMC3990489; doi:10.1371/journal.pcbi.1003568)
Supplement: Figure S4 — A) Individual viral load (drug-sensitive+drug resistant virus) B) Individual ratio of resistant virus to total virus shed depending on time after infection. (y-axis is in log scale) In red, patients with resistant virus emerging and in grey patients without resistant virus emerging. Sample of 100 subjects. (DOCX) [file pcbi.1003568.s004.docx]

**Supplementary information**


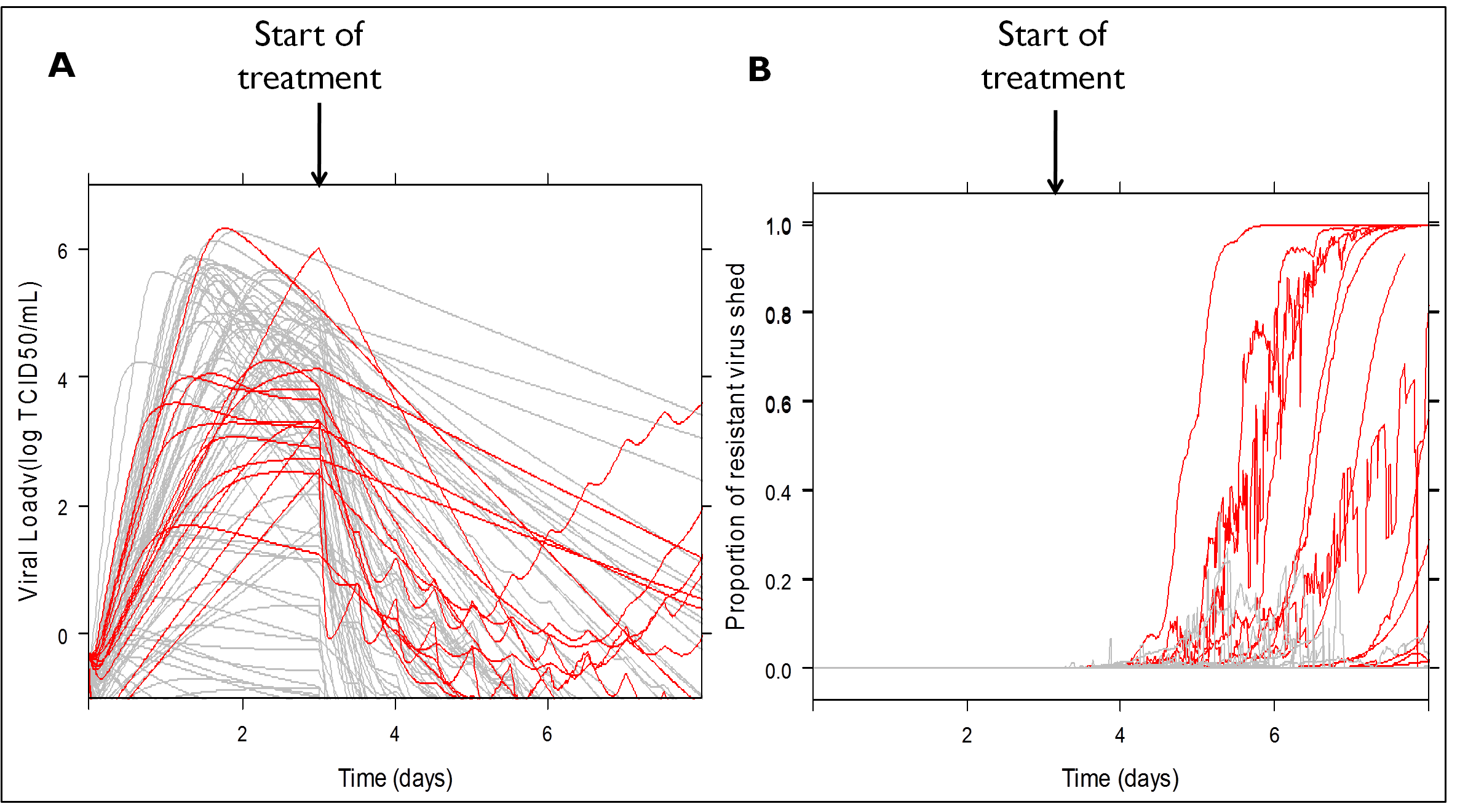


**Figure S4: A) Individual viral load (drug-sensitive + drug resistant virus) B) Individual ratio of resistant virus to total virus shed depending on time after infection.** (y-axis is in log scale) In red, patients with resistant virus emerging and in grey patients without resistant virus emerging. Sample of 100 subjects.
