# Supplementary material for: Impact of Different Oseltamivir Regimens on Treating Influenza A Virus Infection and Resistance Emergence: Insights from a Modelling Study
Source: PLoS Comput Biol. 2014 Apr 17;10(4):e1003568. doi: 10.1371/journal.pcbi.1003568 (PMC3990489; doi:10.1371/journal.pcbi.1003568)
Supplement: Figure S5 — Sensitivity analysis. Comparison, for subjects under 75 mg bid of oseltamivir for 5 days, of the effect of the correction term (panels A to D) for 102 (red), 103 (green) and 104 (blue). Note that we used the mutation rate defined previously (2 106) and comparison of the effect of for the resistant virus (panels E to H) for 0.5 (red), 5 (blue) and 10 (green). Note that we used the mutation rate defined previously (2 106). We also considered in all cases that . (DOCX) [file pcbi.1003568.s005.docx]

**Supplementary information**

**Figure S5: Sensitivity analysis:** Comparison, for subjects under 75 mg bid of oseltamivir for 5 days, of the effect of the correction term (panels A to D) for 10^2^ (red), 10^3^ (green) and 10^4^ (blue). Note that we used the mutation rate defined previously (2 10^6^) and comparison of the effect of $EC_{50}^{S}$for the resistant virus (panels E to H) for 0.5 μM (red), 5 μM (blue) and 10 μM (green). Note that we used the mutation rate defined previously (2 10^6^). We also considered in all cases that $EC_{50}^{R}=200\times EC_{50}^{S}$
