# Supplementary material for: Impact of Different Oseltamivir Regimens on Treating Influenza A Virus Infection and Resistance Emergence: Insights from a Modelling Study
Source: PLoS Comput Biol. 2014 Apr 17;10(4):e1003568. doi: 10.1371/journal.pcbi.1003568 (PMC3990489; doi:10.1371/journal.pcbi.1003568)
Supplement: Figure S6 — Sensitivity analysis 2. Comparison of the effect of the infectivity cost (panels A to D) for 10% (blue) and no infectivity cost (red) and comparison of the effect of for the resistant virus (panels E to H) for 200 (green), 400 (red) and 800 (blue). (DOCX) [file pcbi.1003568.s006.docx]

**Supplementary information**

**Figure S6: Sensitivity analysis 2:** Comparison of the effect of the infectivity cost (panels A to D) for 10% (blue) and no infectivity cost (red) and comparison of the effect of $EC_{50}^{R}$for the resistant virus (panels E to H) for 200 μM (green), 400 μM (red) and 800 μM (blue).
