# Supplementary material for: Impact of Different Oseltamivir Regimens on Treating Influenza A Virus Infection and Resistance Emergence: Insights from a Modelling Study
Source: PLoS Comput Biol. 2014 Apr 17;10(4):e1003568. doi: 10.1371/journal.pcbi.1003568 (PMC3990489; doi:10.1371/journal.pcbi.1003568)
Supplement: Figure S7 — Imperfect adherence. Comparison of the effect of early treatment termination for 75 mg bid (panels A to D) after 10 intakes (red), 6 intakes (green), 4 intakes (blue) and comparison of the effect of early treatment termination for 75 mg qd (panels E to H) after 10 intakes (red), 6 intakes (green) and 4 intakes (blue). (DOCX) [file pcbi.1003568.s007.docx]

**Supplementary information**

**Figure S7: Imperfect adherence:** Comparison of the effect of early treatment termination for 75 mg bid (panels A to D) after 10 intakes (red), 6 intakes (green), 4 intakes (blue) and comparison of the effect of early treatment termination for 75 mg qd (panels E to H) after 10 intakes (red), 6 intakes (green) and 4 intakes (blue).
