# Supplementary material for: Impact of Different Oseltamivir Regimens on Treating Influenza A Virus Infection and Resistance Emergence: Insights from a Modelling Study
Source: PLoS Comput Biol. 2014 Apr 17;10(4):e1003568. doi: 10.1371/journal.pcbi.1003568 (PMC3990489; doi:10.1371/journal.pcbi.1003568)
Supplement: Figure S8 — Effect of conversion factor on individual drug-resistant virus viral shedding. (A) 1 TCID50/mL = 102 virions; (B) 1 TCID50/mL = 103 virions; (C) 1 TCID50/mL = 104 virions; The dashed line represents the limit of detection (LOD) of influenza virus. (DOCX) [file pcbi.1003568.s008.docx]

**Supplementary information**


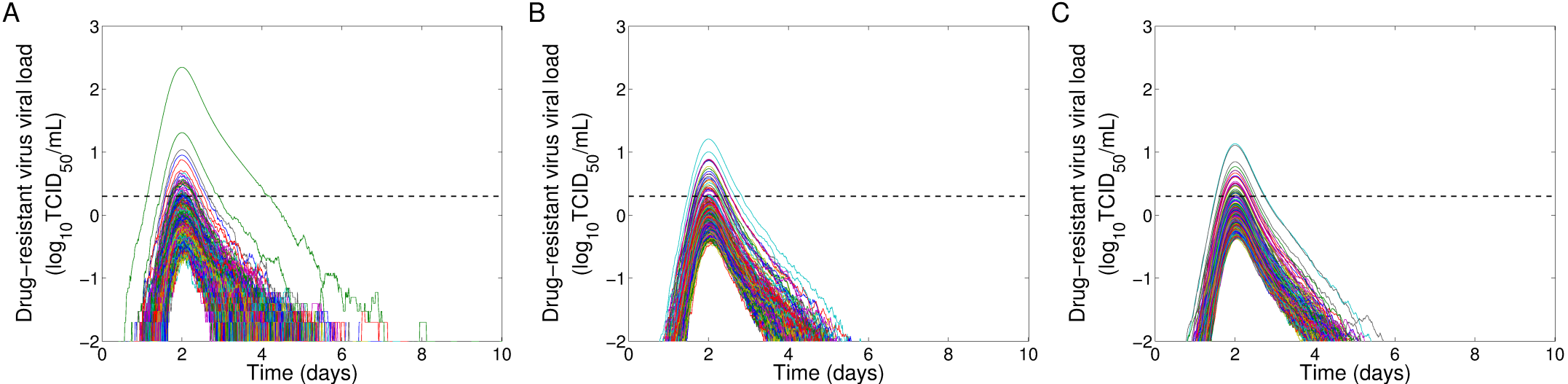


**Fig. S8: Effect of conversion factor on individual drug-resistant virus viral shedding:** (A) 1 TCID_50_/mL =10^2^ virions; (B) 1 TCID_50_/mL =10^3^ virions; (C) 1 TCID_50_/mL =10^4^ virions; The dashed line represents the limit of detection (LOD) of influenza virus.
