# Supplementary material for: Impact of Different Oseltamivir Regimens on Treating Influenza A Virus Infection and Resistance Emergence: Insights from a Modelling Study
Source: PLoS Comput Biol. 2014 Apr 17;10(4):e1003568. doi: 10.1371/journal.pcbi.1003568 (PMC3990489; doi:10.1371/journal.pcbi.1003568)
Supplement: Text S1 — PK model: Analytical solution. (DOCX) [file pcbi.1003568.s009.docx]

**Text S1: PK model: solution**

The PK ODE system can be solved as a sum of three exponentials and used as a time-dependent variable in the VKSD model.

$$\begin{matrix} a_{1}=\frac{\left(ka-ke\right)}{\left(ka-kf\right)}\frac{d\times ka\times kf\left[\exp\left(-ka\times\left(t-lag\right)\right)-\exp\left(-ka\times\left(t-lag\right)+ka\times N\right)dtdose\right]}{1-\exp\left(ka\times dtdose\right)} \\ e_{1}=\frac{\left(ke-ka\right)}{\left(ke-kf\right)}\frac{d\times ka\times kf\left[\exp\left(-ke\times\left(t-lag\right)\right)-\exp\left(-ke\times\left(t-lag\right)+ke\times N\right)dtdose\right]}{1-\exp\left(ke\times dtdose\right)} \\ f_{1}=\frac{\left(kf-ka\right)}{\left(kf-ke\right)}\frac{d\times ka\times kf\left[\exp\left(-kf\times\left(t-lag\right)\right)-\exp\left(-kf\times\left(t-lag\right)+kf\times N\right)dtdose\right]}{1-\exp\left(kf\times dtdose\right)} \\ OC=\frac{a_{1}+e_{1}+f_{1}}{V} \end{matrix}$$

where *lag* stands for the lag-time before oseltamivir phosphate absorption, *ka* is the oseltamivir phosphate absorption rate, *kf* is the oseltamivir phosphate to oseltamivir carboxylate conversion rate, *ke* is the oseltamivir carboxylate elimination rate and *V* is oseltamivir carboxylate volume of distribution. *d* stands for the dose, *dtdose* for the fixed interval between doses, *N* for the number of the dose.
