# Supplementary material for: Impact of Different Oseltamivir Regimens on Treating Influenza A Virus Infection and Resistance Emergence: Insights from a Modelling Study
Source: PLoS Comput Biol. 2014 Apr 17;10(4):e1003568. doi: 10.1371/journal.pcbi.1003568 (PMC3990489; doi:10.1371/journal.pcbi.1003568)
Supplement: Text S2 — Population approach. (DOCX) [file pcbi.1003568.s010.docx]

**Text S2: Population approach**

The population approach is a method commonly used in pharmacokinetics modeling to take into account several levels of variability, such as the inter-individual variability [[1](#_ENREF_1)]. In the population approach, each model coefficient can be decomposed into a “population” parameter (a fixed effect) and an inter-individual variability (IIV) parameter (a random effect).

Let $y_{i}$ be the vector of the $n_{i}$ observations of subject $i$, with $i$varying from 1 to$N$. Let $f$ be a function describing the phenomenon to model (here our structural equations), depending nonlinearly from $\theta_{i}$, the vector of the p parameters of the subject $i$. Suppose that $\xi_{i}$ represents the vector of times at which samples are collected from subject $i$, $\xi_{i}=\left(t_{i_{1}},t_{i_{2}},...,t_{i_{N}}\right)$. The statistical model for the subject $i$ is then:

where $\varepsilon_{i}$ is the vector of the residual errors which is the part of the observations unexplained by the model $f$. We will assume that these errors are independent from one observation to another and that their distribution is Gaussian. $\sigma_{\mathrm{int}er}$ and $\sigma_{slope}$are two parameters characterizing the error model variance.

In the population approach, the structural equation model is common to all the subjects, but the vector of parameter $\theta_{i}$ for the subject $i$ may vary from one subject to another. The vector of parameter $\theta_{i}$ for the subject $i$ can be expressed as a second level model.

Individual parameters were simulated as $\theta_{i} =\theta_{pop} e^{\eta_{\theta i} }$, where θ_i_ is the individual parameter for the subject $i$, θ_pop_ and $\eta_{\theta_{i}}$ are the population parameter and the random effect. $\theta_{pop}$and $\omega_{\theta}$ were drawn from lognormal distributions $\ln N\left(\mu_{\theta},\sigma_{\theta}^{2}\right)$ and $\ln N\left(\mu_{\omega},\sigma_{\omega}^{2}\right)$ respectively, where $\mu_{x}$ and $\sigma_{x}^{2}$ were the mean and variance estimated in previous works [[2](#_ENREF_2),[3](#_ENREF_3)]. $\eta_{\theta_{i}}$ was drawn from a Gaussian distribution $N\left(0,\omega_{\theta}^{2}\right)$, where $\omega_{\theta}$ represents the inter-individual variability of the parameter $\theta$.

**References**

1. Sheiner LB, Steimer JL (2000) Pharmacokinetic/pharmacodynamic modeling in drug development. Annu Rev Pharmacol Toxicol 40: 67-95.

2. Canini L, Carrat F (2011) Population modeling of influenza A/H1N1 virus kinetics and symptom dynamics. J Virol 85: 2764-2770.

3. Wattanagoon Y, Stepniewska K, Lindegardh N, Pukrittayakamee S, Silachamroon U, et al. (2009) Pharmacokinetics of high-dose oseltamivir in healthy volunteers. Antimicrob Agents Chemother 53: 945-952.
