# Supplementary material for: Impact of Different Oseltamivir Regimens on Treating Influenza A Virus Infection and Resistance Emergence: Insights from a Modelling Study
Source: PLoS Comput Biol. 2014 Apr 17;10(4):e1003568. doi: 10.1371/journal.pcbi.1003568 (PMC3990489; doi:10.1371/journal.pcbi.1003568)
Supplement: Text S3 — Probability of extinction. (DOCX) [file pcbi.1003568.s011.docx]

**Text S3: Probability of extinction**

Our results show that long-term (10 or 15 days at time, initiated before inoculation) prophylaxis is very effective (virological and symptom efficacy > 99.9% (see Figure S2). The virological efficacy is not quite perfect however, because of a small fraction of patients who shed resistant virus (proportion of subject shedding resistant virus and proportion of virus shed that is resistant <2.5%, see Figure S3). Taken together we conclude that any symptomatic influenza cases in patients on long-term prophylaxis are likely resistant cases, either through direct transmission or *de novo* mutation. Increased dosing in these patients may more rapidly eliminate the circulating sensitive virus, but would be ineffective against the resistant infection.

These observations are supported by mathematical predictions as discussed below. Near the time of exposure it is reasonable to assume (1) that the innate immune responses are negligible and (2) the number of susceptible cells is approximately constant. Under these conditions, the probability that the infection goes extinct is [[1](#_ENREF_1),[2](#_ENREF_2)]

$$P_{ext}=\left(\frac{\delta\left(c+\beta T_{0}\right)+cp\left(1-\varepsilon_{ss}\right)}{\left(1-\varepsilon_{ss}\right)p\left(c+\beta T_{0}\right)}\right)^{V_{0}TCID}$$

where $V_{0}$is the size of the drug-sensitive inoculum, with TCID the conversion factor from TCID_50_/mL to number of virions and $\varepsilon_{ss}$ the average drug efficacy. Given our population parameters, the probability of stochastic die-out, $P_{ext}$, is 1.0 (IQR: 1.0-1.0) for $V_{0}$=(0.5*TCID_50_/mL,1*TCID_50_/mL,10*TCID_50_/mL). Our model predicts that it is very unlikely that an individual on long-term prophylaxis will become infected by a drug-sensitive strain. However if the inoculum is drug resistant, the probabilities of stochastic die out are 0.48 (IQR: 0.31 – 0.68), 0.11 (IQR: 0.03-0.32) and 0.00061 (IQR: 8.1 10^-6^-0.022) for drug-resistant $V_{0}$=(1, 3 and 10 virions). Taken together our model predicts that, should an individual on long-term prophylaxis develop a symptomatic infection, that infection arose with high probability from transmission of a drug-resistant strain.

**References**

1. Conway JM, Konrad BP, Coombs D (2013) Stochastic Analysis of Pre-and Postexposure Prophylaxis against HIV Infection. SIAM Journal on Applied Mathematics 73: 904-928.

2. Pearson JE, Krapivsky P, Perelson AS (2011) Stochastic theory of early viral infection: Continuous versus burst production of virions. PLoS computational biology 7: e1001058.
